# Implementing injury prevention strategies in community-based youth football: The role of parents, coaches, and organizational leaders

**DOI:** 10.1371/journal.pone.0322373

**Published:** 2025-05-30

**Authors:** Jillian E. Urban, Kimberly D. Wiseman, Justin B. Moore, Madison E. Marks, Ty D. Holcomb, Braydon B. Lazzara, Christopher M. Miles, Laura A. Flashman, Joel D. Stitzel, Kristie L. Foley

**Affiliations:** 1 Department of Biomedical Engineering, Wake Forest University School of Medicine, Winston-Salem, North Carolina, United States of America; 2 Virginia-Tech, Wake Forest University School of Biomedical Engineering and Sciences, Winston-Salem, North Carolina, United States of America; 3 Department of Social Sciences and Health Policy, Wake Forest University School of Medicine, Winston-Salem, North Carolina, United States of America; 4 Department of Implementation Science, Wake Forest University School of Medicine, Winston-Salem, North Carolina, United States of America; 5 Department of Epidemiology & Prevention, Wake Forest University School of Medicine, Winston-Salem, North Carolina, United States of America; 6 Division of Public Health Sciences, Wake Forest University School of Medicine, Winston-Salem, North Carolina, United States of America; 7 Department of Family and Community Medicine, Wake Forest University School of Medicine, Winston-Salem, North Carolina, United States of America; 8 Department of Neurology (Neuropsychology), Wake Forest University School of Medicine, 1 Medical Center Blvd, Winston-Salem, North Carolina, United States of America.; King Faisal University, SAUDI ARABIA

## Abstract

The objective of the study was to gather perspectives and experiences of parents, coaches, and organizational leaders surrounding safety in youth football as it relates to roles and responsibilities of the coach. Parents (n = 13) and coaches (n = 10) of two youth football teams participated in separate, team-specific monthly focus groups to gather their perspectives and experiences surrounding youth football safety. Six organizational leaders participated in one-on-one interviews. Focus groups were coded in Atlas.ti. Interviews were summarized using methods of rapid analysis. Data from focus groups and interviews were integrated and analyzed for thematic content. Parents, coaches, and organizational leaders regarded the youth football coach’s role as “so much more than football,” often serving as role models, mentors, and father figures to athletes. Parents place trust in their son’s coaches and expect them to have knowledge and skills necessary to coach football and teach proper skills to prevent injuries. Organizational leaders set expectations of coaches but recognized the coaches’ autonomy in determining team activities and responsibility for safety. Coaches who teach techniques that are not aligned with current practices and coaches who prioritize winning over safety were identified as concerns for safety. Results demonstrate the important role coaches play in the personal and technical skill development and safety of youth football players and should be considered in the development and implementation of evidence-based strategies to improve safety in community-based sports.

## Introduction

American football is a popular sport with nearly 3 million annual youth participants; however, concern for athlete safety in this popular sport is growing [1,2]. Across all youth sports, football has the highest rate of overall injuries and high risk of concussion [3,4]. Injury prevention initiatives have been implemented from youth through professional levels of football [2,5–7]. Some youth football governing bodies have introduced measures to reduce contact and concussion risk in practices and games [2,8,9]; however, rules, regulations, and oversight vary widely across organizations, leagues, and governing bodies.

The presence of a coach is a common denominator across all of youth football. Coaches play a critical role in the instruction and safety of athletes across all levels of play. Existing interventions emphasizing instruction of proper tackling form through targeted drills, helmetless tackling, and video and verbal feedback on tackling performance have been effective in reducing the frequency and/or magnitude of head acceleration events experienced by athletes, and thus concussion risk [10–15]; however, sustainability hinges on proper training and instruction provided by the head coach and his/her coaching staff [16,17]. In youth sports, coaching and organizational leadership is often provided by volunteers [18], making implementation, enforcement, and accountability of safety rules, regulations, and other interventions a challenge [16]. The Centers for Disease Control (CDC) and USA football have developed educational resources for youth coaches, including signs and symptoms of injury, and coaching certification in safe practices; however, community-level inequalities and challenges have been observed in the adoption of these programs, possibly due to barriers faced by a volunteer workforce [19,20].

Coaches are critical to the implementation of injury prevention programs in youth sports [20–23]. Volunteer coaches vary in attitudes, capabilities, and motivation in coaching and safety domains [24,25], which may influence safety norms, behaviors among athletes, as well as implementation of safety programs [16,26–28]. Sarmiento et al found youth football coaches were receptive to concussion safety changes but believe that these changes limit their ability to create a gameplay environment [10]. Stakeholders in the social and organizational environment (parents and organizational leaders) have a shared responsibility in the youth sport environment, including safety [29]. Kerr et al evaluated sport culture and communication among interscholastic middle school athletes, parents, and staff to understand implementation of injury and concussion prevention. Participants recognized the challenges of prioritizing safety at this unique level of play, and authors recognized the need to create tailored approaches to injury prevention in youth sport settings [30].

How best to design and implement feasible and effective interventions for coaches in this context are not well-understood. Many sports interventions are developed and informed by scientific-evidence, but often include limited or no engagement of end-users to understand their needs and concerns or barriers and facilitators to interventions they are expected to implement. Parents, coaches, and organizational leaders are at the heart of youth football organizations [10,31,32]. Therefore, the study objective was to gather the essence of perspectives and shared experiences of parents, coaches, and organizational leaders surrounding safety in youth football as it relates to roles and responsibilities of the coach.

## Materials and methods

### Study sample

Ten coaches of 12U (i.e., ages and 12 and under as of July 31 for the given season) and 13U (i.e., ages 13 and under) level youth football teams (12U: n = 3, 13U: n = 7) and thirteen parents with athletes on these teams (12U: n = 7, 13U: n = 6) participated in separate, group-specific (i.e., by team and participant type) focus groups at up to four time points. The teams participating in this study were selected due to their participation in a parallel observational biomechanics study [33,34]. Six organizational leaders from different organizations within the same league participated in a single one-on-one interview. Organizational leaders served various roles within their organizations, including president and athletic/football director. This study was approved by the Wake Forest University School of Medicine Institutional Review Board (IRB: IRB00067187) and written informed consent was obtained. Recruitment began 8, August 2021 and ended 19, September 2021. Parents and coaches were invited to participate in focus groups via email and were consented via in person. Organizational leaders were invited to participate via email and consented via e-consent according to IRB protocols.

### Development of data collection materials

Separate group-specific semi-structured guides were developed for each discussion, with input from the research team. The study followed a phenomenological research design to gather the essence of the shared experiences of coaches, parents, and organizational leaders involved in youth football. Experiences and perspectives of being a youth football coach or parent were discussed at the first focus group. This guide was developed to, first, understand the individual’s connection to football and perceived benefits of the sport. Additional questions sought to understand the role of the youth coach in their son’s life and the relationship with their son’s coach among parents. Parallel questions were asked among the coaching groups, including the role they perceive they play in their athletes’ lives and relationship with parents. Both groups were asked about elements of safety discussed among parents and coaches, and specific safety concerns for athletes. Sample questions include, “What role does your son’s coach play in his life?/ What role do you play as a coach in your athletes’ lives?”; “What are your concerns about your child’s health and safety playing football?/ What are your concerns about your athletes’ health and safety on the field?”.

To further dive into perceptions of contact in football, the second focus group involved discussion and presentation of video of football collisions and head acceleration data; parents and coaches shared their perceptions of contact and reflected on the data (e.g., “How do your sons/ athletes describe the contact they deliver/receive in practices or games?”; after viewing video clip, “How would you describe the severity of that hit, or how hard that hit seems, compared to what you might see in a typical practice or game?”). Perceptions of head acceleration data will be presented elsewhere. The topics of the third focus group differed for coaches and parents based on group-specific expressed desire to learn or discuss the respective topics at the next focus group; the coach discussion was focused on practice drills and coaching football technique (e.g., “Describe how you have structured your practice sessions this season.”) while the parent discussion was focused on concussions and clinical implications of subconcussive head impacts (e.g., “What do you think are the risks of not reporting a concussion?”). Finally, the fourth discussion was a focus groups for coaches and interview for parents and was focused on review and discussion of head acceleration data collected during the season (e.g., “How do you think coaches/ you could these data to improve safety or performance?”). The first, second and third focus groups with parents and coaches were one hour and conducted in person. The fourth discussion was conducted in an online format. Organizational leader interviews focused on experiences and perspectives as a leader of a youth football organization following a parallel structure to that of the parents and coaches (e.g., “What role do coaches play in your athletes’ lives?”; “As an organizational leader, what are your concerns about your athletes related to safety on the field?”). All interviews with organizational leaders were approximately 30 minutes and conducted in an online format. Questions were developed to be open-ended. Guides for the focus groups and interviews relevant to the codes and themes evaluated in this study are provided in the supporting information (S1–S6 Files).

Focus groups and interviews were conducted by a single trained member of the study (Jillian Urban, PhD, MPH, Assistant Professor, female). Dr. Urban was trained and mentored in qualitative methods, including focus group and interview moderation, including active listening and effective probing to mitigate bias, such as avoidance of leading questions. Dr. Urban had 9 years of experience working with this youth football community but had no relationship with any of the participants prior to the study. The participants enrolled in this study were familiar with Dr. Urban and the research team through an observational biomechanics study that was conducted in parallel with the focus groups. The participants were acquainted with Dr. Urban’s role at Wake Forest University and prior experience working with the league to study concussions and head impacts in youth football [33,34]. The participants also learned that Dr. Urban was a parent of twins. The participants were made aware that the research team sought to work collaboratively with a set of stakeholders in the local youth football community to create and test a practice structure to reduce head impact exposure while developing the skills needed to play football effectively and safely. They also understood that to inform that effort, the research team wanted to learn more about the perspectives of parents and coaches about football, while sharing some of the data collected in later focus groups. This information was provided at the start of each focus group to recognize potential bias, but also to facilitate trust among the group.

### Data collection and analysis

Focus groups were conducted at a quiet location near the football practice facilities, such as a community conference room or ramada. No other non-participants were present or in close proximity of the focus group. Guides were not provided to participants prior to the discussion. Focus groups and interviews were recorded; a note taker was present to gather detailed notes according to the semi-structured guide. Audio recordings of focus groups and interviews were initially transcribed using an automated transcription application (Otter.ai). A member of the research team listened to the audio and made any necessary corrections to the transcription. Concurrent with data collection, interviews and focus groups were monitored and reviewed for emerging themes by the principal investigator, and it was determined that no additional focus groups or interviews were needed to ensure saturation of the data. A combination of deductive and inductive coding was completed to develop a codebook specifying the code, code definition, exclusion criteria, and exemplar quote(s). Additional codes were added during qualitative coding as new topics emerged. The codebook is provided in the supporting information (S7 File). Two members of the research team independently coded each transcript using the Windows version of ATLAS.ti 22.0.6.0 [35]. The research team members met to discuss coding and resolve discrepancies. Once coding was completed, code reports were run for each code.

Rapid assessment process (RAP) was used to analyze interviews with organizational leaders [36–38]. Domains corresponding to individual questions and in accordance with the order of the guide were developed into a matrix. A member of the research team listened to the audio recordings and completed the matrix in an organized and thorough manner, capturing the main points of the discussion and exemplar quotes. Data in the matrix were organized by respondent and domain for concurrent analysis of all responses [39]. A second member of the research team reviewed the matrix and summary by domain to identify and address any gaps in the summary. This was an iterative and team-based approach that occurred after qualitative coding of the focus groups was complete. The team members then reviewed the matrix by domain, assigned codes from the code book, and annotated additional themes that emerged from the organizational leaders.

Focus groups code summaries and RAP codes from interviews were integrated and analyzed for thematic content by two individuals from the study team [40]. Themes were cross-tabulated with the associated code(s) supporting that theme and indication of the participant group who endorsed that theme. Exemplar quotes were identified for each theme. A wide range of themes emerged from the analysis; however, the current paper focuses on themes related to role and responsibilities of the coach. Similarities and differences across parents, coaches, and organizational leaders are summarized below.

## Results and discussion

Most participants were between ages 35 and 44; most were self-reported to be Black or African American across all participant groups. Most parents were female, while all coaches and all but one organizational leader were male. Demographic characteristics are provided in Table 1.

**Table 1:** Demographic characteristics of parents, coaches, and organizational leaders.

Characteristic	Parents (n=13)	Coaches (n=10)	Organizational Leaders (n=6)
Age; *n* (%)			
<18		1 (10)	
18-24			
25-34	4 (31)	2 (20)	5 (83)
35-44	7 (54)	6 (60)	
45-54	1 (8)		1 (17)
55-64		1 (10)	
Unknown	1 (8)		
Sex; *n* male (%)	3 (23)	10 (100)	5 (83)
Race; *n* (%)			
Black or African American	6 (46)	6 (60)	4 (67)
White	4 (31)	4 (40)	2 (33)
American Indian/Alaska Native	1 (8)		
Other	1 (8)		
Unknown	1 (8)		
Ethnicity; *n* (%)			
Hispanic/Latino/Spanish origin	2 (15)		
Unknown	(8)		

### Roles and expectations of coach

Below is a summary of themes related to roles and expectations of youth football coaches. Exemplar quotes are provided in Table 2.

**Table 2:** Themes and exemplar quotes regarding the roles and expectations of coach.

Theme	L	C	P	Exemplar Quotes
Coaches are considered role models and mentors to athletes, and sometimes serve in a father figure role.	✓	✓	✓	“It depends on the coach, you know. We have some coaches that are out there for two hours. They coach football and have that role of just specifically coach, you know – teaching them the game of football – the x’s and o’s – and they’re great! We have some coaches that are like surrogate fathers. They pick them up, they check on them at school, you know. They make sure they… they help the parents out with funds, unfortunately. They take on a big responsibility and they play a big role in those kids’ lives.” (Leader 3) “Some kids do not have the best role models at home, so coaches show interest in them, how school is going, how life is going.” (Leader 6) “Hopefully [they see me] as a positive black male figure in their lives to see some type of something positive. Where it’s not... We’re not a rapper, or you’re not a football player… it’s not LeBron, it’s not some big glamorous thing. It’s just a regular old person, exuding positivity beyond football is what I think.” (Coach 1) “Some of the kids out here, they don’t have fathers in the home so it’s definitely a way, you know, they look up to the coach, as a father, and as that disciplinarian. So, I mean I think that’s another great thing about the team sport.” (Parent 5)
Coaches recognize their role in developing the skills of athletes in football but feel responsible for also developing athletes as young men and to be “better human beings” off the field.		✓		“For me, it is just to give impact to the youth. I would rather see kids out here trying to learn something and better themselves, get them ready for the next level… but if not, just make sure they’re better men when the time rises.” (Coach 5) “We’re not developing just players, like he said, we’re developing young men too.” (Coach 2) “My biggest thing is as a coach: I don’t care about wins; I don’t care about losses. What I care about most is these guys take what we teach them on the field and transpose that into life being better human beings. And so, the only way they can do that is they learn how to be disciplined on the football field, they give effort, and don’t quit on themselves... So, what can I do mentally to prepare them for next level, not just in football, but off the field as well.” (Coach 4)
Parents place trust in their son’s coaches and expect them to have the knowledge and skills necessary to coach football.			✓	“And so, we have always kind of said, like, we’re going to make sure that you’re on a team where the coaches are doing the right thing and know what they’re doing.” (Parent 4) “Just being safe. Yeah, making sure the coaches are doing things that will teach them football but also keep them protected.” (Parent 3) “My son… he different. He thinks he’s Superman so I just don’t argue with him about none of that. So, if he get hurt, he’ll let me know and we’ll take care of that, but not to be scared. Like I tell him, if you do what coach tell you what to do and most the time, if you do it the right way, you won’t get hurt.” (Parent 10)
Coaches must manage relationships with parents and athletes to successfully coach their team.	✓	✓		“No matter how great a coach you are. No matter what position your team is in… there’s still parents who are not happy. And there’s nothing we can do about it.” (Leader 5) “And you have parents who think their child is Ray Lewis. I mean, every father and mother... Every parent believes that their child is a little better than they actually are, no matter how good the child is. If the child is great, they think he’s greater. If he’s not good at all, they think he’s decent. I mean, it’s just the way it is. So, you have to… you have to let... I mean [coaches] just have to put it out there, you can’t hide it. Um Yeah, gotta see where everyone is.” (Coach 1) “But, you know, and, you know, just building the relationships with, with kids. You know, that it’s... it’s funny how 12-year-olds respond to people who treat them like people. So, we try to talk to them and treat them like young men. And, you know, there’s discipline that’s involved a lot of times, of course they’re 12, but you know, that’s our philosophy is we coordinate as a group and the players, I think, understand that we’re all on the same front when we bring something to them.” (Coach 3)
Organizational leaders help manage relationships with parents, athletes, coaches, etc.	✓			“I tell [coaches] their job is to take care of the football team. My job is to take care of the organization, the parents, and I am also that buffer.” (Leader 1) “I try to let the coach handle it at first... but I like to be around or be close and that’s for the coaches’ protection, as well... I need the coach to know… because I’m usually harping on them about something… that I also have his back in decisions and so sometimes, you know, I’ll be there when the interaction happens just as a third party, just, you know, so on both sides, you know, I’m neutral. I don’t have affiliations to one particular team, you know. I’m with the whole, our whole organization.” (Leader 3)
Coaches foster the child’s continuation in the sport of football.	✓	✓	✓	“I tell the flag coaches, you know, you’ve got to build the organization. Because if you do your job right, then the kids will return, and they’ll want to play football next year. With the tackle coaches, you know, I just stress the fact that my philosophy is I don’t believe in minimum play players. I don’t believe any kids should just get their six or eight, whatever the case may be, and sit on a bench. And that’s one of the things I talk to all my coaches about is making sure the kids stay involved in the game. You know, it all basically boils down to all coaches creating a positive experience for the kids in our program.” (Leader 5) “You got coaches out here that do whatever it takes to win football games. And so, our coaching staff, we’ll take in any kid that we can possibly get. You got coaches out here who will stack a football team, get every best kid they possibly can to go win a national championship for their glory sake, but when a kid turns to high school, they have no fucking clue what they’re doing. And now you wasted that kid’s ability to teach them at this level, to transition them to high school, to actually be somebody, but now they’re literally lost in the sauce and can’t compete for a position. Now they lost the love of the game, and also put themselves in a position to get hurt also as well.” (Coach 4) “Number one is coaching. A good coach that’s there for the right reasons to teach my child how to get to that next level is very important.” (Parent 11)

Checkmarks indicate the participant group (L = Leader, C = Coach, P = Parent) that endorsed each theme.

*Coaches are considered role models and mentors to athletes, and sometimes serve in a father figure role*. The role of the coach in the athletes’ lives was regarded as “so much more than football.” Respondents remarked the different ways that coaches support athletes, including providing transportation to practices and games, holding them accountable for and helping them with academics, helping them navigate social situations, guiding them in making good choices, teaching them respect for authority, and sometimes providing financial support. Organizational leaders recognized that coaches have a more hands-on role with athletes than organizational leadership. Several coaches, some organizational leaders, and one parent noted that coaches supplement mentoring that may be absent from the athlete’s home environment. One organizational leader acknowledged that some coaches are present to strictly coach football, while others are like “surrogate fathers.” Five coaches discussed their role in helping raise the athletes. Mentorship provided by coaches was regarded as a benefit of youth football.

*Coaches recognize their role in developing the skills of athletes in football but feel responsible for also developing athletes as young men and to be “better human beings” off the field through the sport of football*. Many coaches described their role in teaching skills of football and proper techniques to prevent injuries; many described this as teaching athletes how to protect themselves and “getting them ready for the next level.” Coaches also described their role in teaching life skills, including discipline, dealing with adversity, and being resilient. Some coaches described their philosophy around developing athletes for life off the field, including holding athletes accountable for their behavior and school performance and developing skills of hard work and resiliency. Many coaches regarded the lessons learned playing football are lessons that benefit adolescents in ‘real life’. One coach described his role in encouraging healthy behaviors like staying hydrated and eating nutritious foods.

*Parents place trust in their son’s coaches and expect them to have the knowledge and skills necessary to coach football.* Nine parents expressed an expectation that their son’s coaches are knowledgeable and skilled in coaching football, expecting coaches are doing the “right thing” and know what they are doing. A subset of those parents expect coaches will teach their son skills to keep themselves protected and safe while playing the sport. Five parents discussed the expectation that coaches have the appropriate training necessary to coach youth football. Some parents discussed a desire for coaches to be willing to learn and make changes if needed.

*Coaches must manage relationships with athletes and parents to successfully coach their team.* Coaches and some organizational leaders noted the importance of communication in coaching. One coach described his communication style as ‘tough love’, stating that he often yells at the athletes to communicate his message, whereas another coach described the importance of talking with them like young adults and building trust. One organizational leader recognized that coaches must adapt to different communication and learning styles of athletes. Coaches also described their expectations of athletes, including time, effort, confidence, leadership, and willingness to be coached. About half of the coaches discussed their philosophy around working together as a coaching staff but noted the importance of the head coach as the leader. Coaches also discussed the need to manage expectations of parents around their child’s abilities and how the coach utilizes the child on the team (position, playing time, etc.). One coach discussed sharing film as a means to help manage expectations. Organizational leaders, however, noted the important role coaches play in making sure parents and athletes have the “best experience possible” participating in football.

*Organizational leaders help manage relationships with parents and coaches.* Organizational leaders oversee the organization and provide support for the coaches and families within. Many organizational leaders described their role in mediating relationships, often acting as a “buffer” or “third party” between parents/families and coaches. Organizational leaders spoke about common conflicts among parents and coaches, including parent expectations surrounding their son. This included the perceived skill level, allotted playing time and/or number of plays their son gets the ball, and playing position chosen by the coach. Organizational leaders described how they try not to intervene, unless necessary, and described their role in supporting their coaches but listening to parent concerns to address or rectify any issues.

*Coaches foster the child’s continuation in the sport of football*. Organizational leaders described the coaches’ role in developing the child’s enjoyment of football and identified coaches as critical to the success and well-regard of the organization. One coach and one parent noted the coaches’ role in creating a positive environment for the athletes and another coach remarked that an overemphasis on winning by coaches can ruin a player’s love for the game.

### Practice and safety

Below is a summary of themes related to practice and safety. Exemplar quotes are provided in Table 3.

**Table 3:** Themes and exemplar quotes related to practice and safety.

Theme	L	C	P	Exemplar Quotes
Practice is the “foundation” for athlete development and important for teaching athletes the fundamentals of football, including technique, safety, and responsibilities for a game.		✓	✓	“I mean, [practice is] pretty much the foundation. If you ain’t at practice, you ain’t learning and it’s obvious. I mean, you’ve got to be there, from tackling to how to line up, I mean, everything is pretty much around practice. And it shows, the kids that show up for practice are the kids normally, even if they’re not great athletes, they at least show that they’re developing.” (Coach 7) “[Practice is] everything, everything, because that’s where you essentially build the foundation of it also. I mean, it kind of affects everything.” (Parent 6) “You don’t practice, you’re not gonna do it in the game.” (Parent 9) “So they won’t be scared. They know what they looking for.” (Parent 8) “And know what to expect and know how to react next time I get hit.” (Parent 11) “Well, and a lot of game play is reaction, so if you’ve practiced it the right way, maybe you’ll be more likely to do it the right way in a reaction setting instead of just throwing yourself at somebody.” (Parent 4)
Coaches use drills in practice to teach the fundamentals of football and to assess and develop athlete skills in practice.		✓		“So we try to prepare our boys around that situation we’re going to play, and those scrimmages are catered towards the plays, we’re going to run for Saturday. And so we have a script, we have different defensive calls that we want to run, we try to put kids in position. Our biggest thing is, if you’re in position to make a play – make a play, and sometimes they do it, sometimes they do their own things, we can’t... it’s out of our control. But scrimmages help us get ready for game day on Saturday.” (Coach 4) “Mano y mano, who wants it more?...When you’re playing when you’re playing on Saturday, you know, any day… Thursday, Friday, Saturday, Sunday, you know, whatever level you play when you line up it’s me versus you. And my job is to beat you. Before I can do anything else so like, that’s our one on one drills of stressing the fact that you have to do your job and right now he’s your job. And if you don’t beat him right now. How can we assume or trust that you’re gonna beat that person on Saturday? Right now, so it’s really, that’s our probably our most physical drill is the, you know, it’s not tackling to the ground but it’s the one on one hand fighting the dog eat dog in the trenches.” (Coach 2)
Organizational leaders expect coaches to stay up to date on current practices related to safety and give them autonomy over their team but recognize that they need to hold the coaches accountable in using safe practices.	✓			“I tell them their job is to take care of the football team. My job is to take care of the organization and the parents… I do try to stay hands off, you know. I want it to be their team.” (Leader 1) “We oversee the football and the cheer, as well as the coaches – making sure they are in compliance and that they take their required tests for certification to coach and work with youth kids.” (Leader 2) “I can be like an advisory, and also the hall monitor. So, I make sure they’re doing the right thing because I know sometimes, they can get sidetracked. My coaches… I let them kind of run their own program, as long as their doing it safely. And if I see a drill that I know is outlawed, I’ll pull them to the said and say, ‘hey coach, you shouldn’t be doing that.’ I don’t bark orders at them but I make sure everybody’s on the right path.” (Leader 3)
Coaches are responsible for the safety of the athletes on their team and are expected to teach athletes proper skills and techniques to prevent injuries.	✓	✓	✓	“So the coaches play a major, major role and we want to make sure that they are up to date with the proper techniques on coaching the kids so that they won’t get injured. And then they can move forward from that point.” (Leader 2) “It’s about protecting them and teaching them how to protect themselves.” (Coach 3) “That’s our main job, you know, teach them technique, protect themselves, and getting them ready for the next level. And if we can win along the way… You know, coach used to tell me even, one of my mentors, if you develop players, teach them proper technique, skill, if you develop players, winning takes care of itself.” (Coach 2) “I think it’s the coach’s responsibility to teach these kids the proper techniques to keep people from getting concussions and not to lower their head. I mean, just... there’s all kinds of different stuff that... I do put it back on the coaching and it is part of the coaching that that influences these kids. There’s good coaching and there’s bad coaching, that’s everywhere. As far as what I would let them... I mean, I really wouldn’t know other than other than that, I just feel like it falls back on the coaching.” (Parent 11)
Coaches who teach players techniques that are not aligned with current practices and coaches who prioritize winning over safety were identified as concerns for player safety.	✓	✓	✓	“My primary concern is basically… I know what our organization… how our organization teaches things. And I know what our kids are taught or how our kids are taught to play football and how our kids are taught to tackle. But, it’s not universal within a league. And there’s always organizations out there where they believe in teaching their kids to do things what I consider ‘the wrong way’. You know, they want that big bang, that big pop – that big ‘oo’ or ‘ahh’ hit and it’s not safe. It becomes an issue for me because, you know, our kids get hit the wrong way and they’re hurt… They’re young children and they shouldn’t be taught to tackle the wrong way. They shouldn’t be taught to do things that could subject them to being seriously injured.” (Leader 5) “It’s not me and [other coach] coaching. What’s the guy on the other sideline coaching? How is he coaching his players? Because I’ve seen a lot of times those kids ain’t being taught the right way. And the kids are getting hurt because they’re not being taught the right way. So that’s just not the coach... that’s on the organization as well to find these coaches who can coach and coach the right way.” (Coach 7) “Also, you got coaches out here that do whatever it takes to win football games. And so our coaching staff, we’ll take in every kid that we can possibly get. You got coaches out here who will stack a football team, get every best kid they possibly can to go win a national championship for their glory sake, but when a kid turns to high school, they have no fucking clue what they’re doing. And now you wasted that kid’s ability to teach them at this level, to transition them to high school, to actually be somebody, but now they’re literally lost in the sauce and can’t compete for a position. Now they lost the love of the game, and also put themselves in a position to get hurt also as well.” (Coach 4)

Checkmarks indicate the participant group (L = Leader, C = Coach, P = Parent) that endorsed each theme.

*Practice is the “foundation” for athlete development and important for preparing athletes for competition.*

All but one coach and all but one parent described the role of practice in preparing athletes for games. Coaches described this preparation as learning the “fundamentals,” including technique, safety, and responsibilities for games. Some coaches and parents felt activities in practice teach athletes to be unafraid of contact and “being confident in being physical,” and many described how practice helps get players acclimated to contact prior to a game; some coaches and parents felt this was important for younger players learning how to tackle.

*Coaches use drills in practice to teach the fundamentals of football and to assess and develop athlete skills in practice*. Coaches discussed drills they commonly use in practice, including scrimmage, angle tackle, one-on-one tackle, and open-field tackle. Five coaches described the purpose of the scrimmage drill as a way to prepare for games and teach strategy, skills, and technique. One coach felt that contact can be limited in scrimmage with “quick whistles”. Three coaches from one team discussed using the angle tackle drill to teach pursuit, running angles, and to reinforce “near foot, near shoulder” – a method of removing the head from the tackle. Two coaches shared their thoughts, with three additional coaches in agreement, on how they provide instruction for drills, including demonstrating the drill, having players walk through the drill, and then executing the drill and using film to show athletes their form. One-on-one style drills such as one-on-one tackle and open-field tackle were discussed by some coaches as intended to stress physicality and one coach noted running these drills because kids are “afraid to tackle”.

*Organizational leaders expect coaches to stay up to date on current practices related to safety and give them autonomy over their team but recognize that they need to hold the coaches accountable in using safe practices*. Organizational leaders described their role in setting expectations of coaches, including completion of required trainings and certifications and following rules of the organization; however, many described their leadership philosophy as “hands off” and expressed their desire to let coaches organize and conduct activities of their team as long as it is done safely. In addition, organizational leaders described their role in maintaining rules of order and proper decorum of coaches, describing their role as “managing the ship”, “hall monitor”, and “coach to the coaches”. Organizational leaders provided examples of when they would need to intervene on coach activities, including unfair abuses of power or authority, unequal opportunities of playing time, unsafe practices on-field, or disrespect for kids and parents.

*Coaches are responsible for the safety of the athletes on their team and are expected to teach athletes proper skills and techniques to prevent injuries*. Many coaches and organizational leaders discussed the coaches’ responsibility in developing skills and techniques of athletes. Strategies for teaching athletes proper skills and techniques described by coaches include demonstrating the drill before players engage in the drill, reviewing the proper way to tackle by having the players practice without helmets, and using film to facilitate discussion around technique. Many coaches and organizational leaders acknowledged that tackling technique has evolved to improve safety as knowledge surrounding concussions has increased. They discussed the risk of injury, including concussion, that comes from coaches not teaching, or players not using, proper technique. Some coaches described the rewards of teaching players proper technique, including preparing for and winning games and reducing injuries. Parents recognized the importance of coaches in the instruction of technique. Parents, particularly moms, described how they tell their sons to “be careful” and provided reassurance to the athletes due to having seemingly knowledgeable coaches and protective equipment. Some parents described their expectation that coaches will teach their sons skills to keep themselves protected and safe while playing the sport. Some parents discussed how technique instructed by coaches can be used to prevent concussions; coaches and organizational leaders echoed this sentiment. One parent mentioned her expectation that coaches are familiar with signs and symptoms of concussion.

*Coaches who teach players techniques that are not aligned with current practices and coaches who prioritize winning over safety were identified as concerns for player safety*. Many coaches discussed the influence of “bad coaching” on players, including coaches who just “want to win” and lack of coaches who “worry about the safety of it.” Organizational leaders acknowledged that some coaches have “ill-advised” aspirations and expressed concerns for the increased risk that poses for others. Some coaches and parents discussed concern for ill-motivation of opposing coaches or lack of necessary training or coaching experience among other coaches and its effect on player safety. Organizational leaders expressed concern for instruction provided by other organizations, including teaching athletes improper techniques, loopholes, and ways to cut corners in order to get a big hit.

## Discussion

This study combines the views of different stakeholders to inform the roles, responsibilities, and impact of coaches on safety strategies in youth American football. Parents, coaches, and organizational leaders recognized the diverse role coaches play in the development and safety of young athletes, including role model, mentor, and father figure. Prior literature has demonstrated that coaches are critical to young athletes’ personal and sport development and can positively or negatively impact an athletes’ experience in sport [41]. Furthermore, others have demonstrated the direct impact coaches have on an athlete’s perception of their performance, motivation, and status on the team [42–45]. Coaches also influence team culture, including perceptions of injuries and risk [43,46,47]. Our findings combined with prior literature support that leagues and organizations must leverage resources to address the dynamic and important role coaches play in athlete development, including mentorship and training specific to coaching young athletes.

The importance of coaches in managing relationships with athletes and parents to successfully coach their team was recognized by organizational leaders and coaches. Coaches discussed unique communication styles, and an organizational leader noted the importance of adapting to the communication and learning style of their athletes. Horn et al further summarizes the importance of coach behavior and communication, indicating that the type and amount of feedback and expectations communicated to the athlete can shape an athlete’s performance, behavior, and motivation in sport [48]. Expectations were a common theme that emerged, where coaches described the need to set expectations among athletes and manage expectations of parents, while ensuring both parties have a good experience in the sport. Santos et al performed a scoping review of parent-coach interactions in youth sport and reported common themes in the literature that support this finding and highlighted the need to create meaningful relationships grounded in open and honest communication between coaches and parents, as parents are an integral part of the team [49]. Parents may have unrealistically high expectations of their children, which can negatively impact the child’s enjoyment and experience in the sport [50,51]; therefore, it is critically important for coaches to set realistic expectations for the team as well as expectations for parent behavior, while maintaining open communication, as recognized by the organizational leaders and coaches.

Parents place trust in coaches and expect coaches will have the knowledge and skills necessary to coach football safely and effectively. Sarmiento et al reinforced this funding in a qualitative study, reporting that parents felt coaches should teach young athletes how to prevent injuries, such as a concussion, by teaching them to “hit correctly” [52]. Youth coaches with prior experience playing and coaching the sport tend to have greater coaching efficacy among parents [53,54]. Other factors that may influence parent perceptions include prior experience, social exchanges in the community [55], evidence of effective verbal and non-verbal communication [56], and/or trust in the league or organization they are enrolled in [53]. Furthermore, lack of trust leads to ineffective practices for parent-coach interactions [56]. Many youth football coaches are parent volunteers and may lack the training necessary to coach athletes appropriately and/or deliver injury prevention programs [16]. Voluntary educational programs have been developed for youth football coaches and community leagues have varying rules on the required experience and training necessary to coach young athletes [57]. Communities with fewer resources may face barriers in adopting educational programs for coaches and community-level inequalities have been observed in concussion education programs for youth football coaches [19]. Notwithstanding the barriers in time, resources, and commitment to complete such training for volunteers, there are also deficits in coaching education to adequately meet the needs and expectations of coaches [17]. Organizational leaders in this study recognized their responsibility in holding coaches accountable in their safety training compliance. Further engagement of coaches and organizational leaders will facilitate our understanding of barriers and facilitators to improving the knowledge and skills of youth football coaches and identify practical and efficient strategies to deliver educational resources for coaches.

The responsibility and expectation of coaches to teach athletes proper skills and techniques to prevent injuries in practice was recognized across participant types. Daniel et al demonstrated that most high-level impacts in youth football occur during practices, not games [58]. Nearly two-thirds of the head impacts a youth football player receives in a single season occur in practice and head impact exposure is influenced by coach-directed activities (i.e., drills) [59–62]. Coaches who teach players techniques that are not aligned with current practices and coaches who prioritize winning over safety increase player safety risk was identified as a common concern. Organizational leaders described the autonomy given to coaches to organize and conduct the activities of practice; however, oversight of safe practice activities without stronger enforcement and accountability at the organizational, league, and governing bodies levels can lead to disparities in use of safe practices. Hornsby et al points out that the autonomy given to coaches assumes that the coaches are appropriately equipped to execute their expected responsibilities [17]. Leagues and governing bodies may need to directly target organizational-, team-, and individual-level barriers to implementing safe practices among coaches in youth football practices [19].

The role of the coach in implementing injury prevention programs has also been identified in youth football, rugby, soccer, and other sports [16,20–23]. In a survey of youth rugby union stakeholders, nearly 85% of parents considered coaches to be responsible for injury prevention programs. Coach attitudes, motivations, and leadership were identified as barriers to implementing the injury prevention program; coach buy in and perception that the program was recognized as a valuable facilitator [32]. Parents of youth football players rely on coaches to communicate with athletes about concussion reporting and recovery [52]. Coaches are expected to play a critical role in disseminating information gained from educational programs (e.g., CDC HEADS UP) about concussions to athletes and influence athlete knowledge of concussion, use of safe practices, and injury reporting behavior [47,63]. Participants in this study also recognized the importance of coaches in the safety and instruction of athletes, and many expressed the expectation that coaches have the knowledge, training, and desire to use safe practices. Coaches are important targets for delivering injury prevention programs but should also be engaged as key stakeholders in the development of programmatic materials to maximize the feasibility, acceptability, and sustainability of such programs. Future studies should also recognize the authority given to head coaches but consider the shared responsibility of assistant coaches and other team resources at the youth level (i.e., team mom, organizational leaders) to cultivate an environment conducive to successful delivery and maintenance of injury prevention programs [25,58].

Numerous participants recognized the father figure role coaches provide to athletes in football, in addition to their coaching responsibilities. Trusted adults outside of the family are important to the development of adolescents [64]. Youth coaches can enable social support and social capital of youth, supplementing the male mentorship that may or may not be present in the home environment for young male football players [64,65], as recognized by the participants in this study. Coaches have the opportunity to be positive male adults, contributing to the community necessary for positive development of youth. While this study did not have the opportunity to explore gender differences in coaching, football is a predominantly male sport, with proportionately high number of male coaches. Future work may explore the role of gender, race, and ethnicity in the roles and responsibilities of coaches.

This study is subject to limitations. The results are based on the authors’ interpretation of qualitative data collected; participants did not have the opportunity to review or reflect on their responses or themes that were identified. Additionally, participants represent a limited sample of parents, coaches, and organizational leaders from a single youth football league operating under guidelines of American Youth Football which limits the generalizability of these findings. The perspectives and experiences of participants may not fully reflect those of the greater population, although similar themes from other studies were identified. These limitations should be considered when interpreting and generalizing findings from this study. The moderator’s guide changed for each meeting, but all groups were analyzed together for this manuscript. The questions in the guides for parents, coaches, and organizational leaders mirrored one another, but questions were worded from the perspective of the participant type. We did not analyze if differences in themes endorsed by each group were due to different opinions or if they were the result of probing strategies of the moderator. Future analysis could evaluate how themes and topics emerged over time or the nature of differing opinions. Organizational leader interviews were conducted virtually. There are limitations to virtual data collection, including technical difficulties and limited ability to read body language. Special considerations were taken to employ robust data collection methods. Finally, given the nature of the topic, participants may have provided socially desirable answers.

This study informs the roles, responsibilities, and expectations of youth football coaches from the perspectives of parents, coaches, and organizational leaders. Results highlight the important role coaches play in the personal and technical skill development and safety of youth football players and expectations of parents and organizational leaders have for youth coaches in knowing and using safe practices. Coaches have autonomy in instructing and coordinating their team, but additional accountability may be necessary to ensure coaches are implementing safe practices. Parents, coaches, and organizational leaders are important stakeholders in community-based youth sports and should be considered in the development and implementation of evidence-based strategies to improve safety in community-based sports. Future work may also consider engagement of youth athletes to understand their perspectives toward safety and coaching as well. The results of this study will inform the development of an evidence-based intervention to reduce concussion risk and head impacts using a community-engaged approach [33].

## Supporting information

S1 File Parent Focus Group #1. Moderator’s guide for the first parent focus group. (PDF)

S2 File Parent Focus Group #4. Moderator’s guide for the fourth parent focus group. (PDF)

S3 File Coach Focus Group #1. Moderator’s guide for the first coach focus group. (PDF)

S4 File Coach Focus Group #3. Moderator’s guide for the third coach focus group. (PDF)

S5 File Coach Focus Group #4. Moderator’s guide for the fourth coach focus group. (PDF)

S6 File League Official (i.e., Organizational Leader) Interview #1. Moderator’s guide for the organizational leader interview. (PDF)

S7 File Evidence-Based Intervention in Youth Football Codebook. Codebook used in this study.
(PDF)
