# Supplementary material for: Implementing injury prevention strategies in community-based youth football: The role of parents, coaches, and organizational leaders
Source: PLoS One. 2025 May 30;20(5):e0322373. doi: 10.1371/journal.pone.0322373 (PMC12124582; doi:10.1371/journal.pone.0322373)
Supplement: S1 File — (PDF) [file pone.0322373.s001.pdf]

Parent Focus Group #1 - Moderator's Guide

Good evening, everyone! Welcome to our first youth football parent focus group. My name is Jill Urban. I am an Assistant Professor at Wake Forest University. I am a parent myself with three-year old twin boys. I have worked with several youth sports organizations, including the league studying concussions and head impacts in youth football for the past 9 years.

We have a new project starting this fall to work collaboratively with a set of stakeholders in the local youth football community to create and test a practice structure to reduce head impact exposure while developing the skills needed to play football effectively and safely. To inform that effort, we would like to learn more about the perspectives of parents and coaches about football, while sharing some of the data collected on field in our later focus groups. I'd like to introduce you to [Madi, Ty, Alexandra, Tina]. He/she is a graduate research assistant, and she will be taking notes today.

What we'll be doing today is having a discussion about your experiences being a parent to a child who participates in youth football. Before we get started, I wanted to state a few ground rules. First, there are no right or wrong answers to my questions. We genuinely want to hear from you so please share your perspectives and experiences, both positive and negative. Please also be respectful of one another. If you have a different opinion than someone, it is ok to share it but please be respectful. Please respect one another's privacy – what is said in this room stays in this room. Additionally, to protect your privacy, we will not be taking notes with names of who said what and we will not discuss what is said in these meetings with other focus groups, parents, or coaches.

Just a reminder - I will be recording this conversation. Please speak clearly and try not to talk over one another. I may ask you to repeat yourself, if needed. Please also try to limit distractions, like cell phones during the meeting.

If you need to leave for any reason to use the restroom or to take a phone call, please feel free to do so.

Okay, so we'll go ahead and get started.

1. What is your personal connection to football?
    a. Have you ever played football?
2. Why does your son play football?
    a. Or why does your son play football, as opposed to another sport?
3. In your opinion, what are the benefits of kids participating in youth football?
    a. Why are you, as a parent or guardian, supportive of your son playing football?
4. What role do team sports play in your son's life?
5. What role does your son's coach play in his life?
6. Tell me about your relationship, as a parent or guardian, with your son's coach.
7. Have you ever discussed football safety with your son's coach [or with other league officials]?
8. What are your concerns about your child's health and safety playing football?
9. What are your hopes and goals for your son playing football this fall?
10. What are you hoping to learn from participating in the focus groups?
11. Do you have any other thoughts related to what we've talked about today that you'd like to share?

Thank you so much for sharing your thoughts and opinions today! Here's the plan moving forward:

We will have three additional focus group meetings during September, October, and November. Our upcoming meeting topics will include common drills and contact scenarios in football, as well as concussions and hits to the head in football. Additionally, we will discuss some of the biomechanics and video data we collect with the sensors on field.

-
