# Supplementary material for: Implementing injury prevention strategies in community-based youth football: The role of parents, coaches, and organizational leaders
Source: PLoS One. 2025 May 30;20(5):e0322373. doi: 10.1371/journal.pone.0322373 (PMC12124582; doi:10.1371/journal.pone.0322373)
Supplement: S2 File — (PDF) [file pone.0322373.s002.pdf]

Parent Focus Group #4 - Moderator's Guide

Good evening, everyone! Welcome back to our youth football parent focus group meetings. My name is Jill Urban. I am a professor at Wake Forest University, and I will be leading our discussion today.

As a reminder, we have a new project to work collaboratively with a set of stakeholders in the local youth football community to create and test a practice structure to reduce head impact exposure while developing the skills needed to play football effectively and safely. To inform that effort, we would like to learn more about the perspectives of parents and coaches about football, while sharing some of the data collected on field. I'd like to introduce you to [Madi, Tina]. She is a graduate research assistant, and she will be taking notes today.

Today, we will be having a discussion about concussions and head impacts in football. Before we get started, I wanted to remind you of our ground rules. First, there are no right or wrong answers to my questions. We genuinely want to hear from you so please share your perspectives and experiences, both positive and negative. Please also be respectful of one another. If you have a different opinion than someone, it is ok to share it but please be respectful. Please respect one another's privacy – what is said in this room stays in this room. Additionally, to protect your privacy, we will not be taking notes with names of who said what and we will not discuss what is said in these meetings with other focus groups, parents, or coaches.

Just a reminder - I will be recording this conversation. Please speak clearly and try not to talk over one another. I may ask you to repeat yourself, if needed. Please also try to limit distractions, like cell phones during the meeting.

If you need to leave for any reason to use the restroom or to take a phone call, please feel free to do so.

I'd like to start today with a summary of the biomechanics data we collected from your team last fall – [15 minutes]

1. Do you have any questions about the data?
2. As a parent, what would be helpful to know about the head impacts and concussions BEFORE the season starts?
3. If you had information about your son's impacts, what would you do with it?
4. How do you think coaches could use these data to improve safety or performance?
5. In your opinion, what is the greatest opportunity to reduce hits to the head in practice youth football?
6. What are the challenges in implementing changes to practice at the youth levels of football?
    a. What are the challenges in enforcing changes to practice at the youth levels of football?
    b. What are the challenges in sustaining changes to practice at the youth levels of football?
7. In your opinion, what would facilitate or motivate coaches or league officials to engage in discussions about changes to practice at the youth level of football?
8. From your perspective, how do you define a successful football season for your son?
9. What are the top three things that contribute to your son's success in football and why?
10. Do you have any thoughts or questions about what we discussed today?
11. What are you hoping to learn at the conclusion of our study?

Thank you so much for sharing your thoughts and opinions for this project!
