# Supplementary material for: Implementing injury prevention strategies in community-based youth football: The role of parents, coaches, and organizational leaders
Source: PLoS One. 2025 May 30;20(5):e0322373. doi: 10.1371/journal.pone.0322373 (PMC12124582; doi:10.1371/journal.pone.0322373)
Supplement: S3 File — (PDF) [file pone.0322373.s003.pdf]

Coach Focus Group #1 - Moderator's Guide

Good afternoon, everyone! Welcome to our first youth football coach focus group. My name is Jill Urban. I am an Assistant Professor at Wake Forest University. I have worked with several youth sports organizations, including your organizations studying concussions and head impacts in youth football for the past 9 years. Thank you so much for your support this season and for encouraging your athletes to participate. I have appreciated the partnership with the league for the last several years, and I can't speak enough to the awesome support we have from our team coaches. I am also a parent myself with three-year old twin boys.

We have a new project starting this fall to work collaboratively with a set of stakeholders, including coaches, players, and officials, in the local youth football community to create and test a practice structure to reduce head impact exposure while developing the skills needed to play football effectively and safely. We collect a lot of data that may be useful for coaches, so we would like to put the data back in the hands of the decision makers to try to create an effective practice environment while minimizing the risks, where possible. To inform that effort, we would like to learn more about the perspectives of parents and coaches about football, while sharing some of the data collected on field in our later focus groups. Many of you know Madi. She is a graduate research assistant leading data collection at the [Team] this fall, and she will be taking notes today.

What we'll be doing today is having a discussion about your experiences and perspectives being youth football coach. I will be asking several questions and facilitating the discussion – none of them are hard. Before we get started, I wanted to state a few ground rules. First, there are no right or wrong answers to my questions. We genuinely want to hear from you so please share your perspectives and experiences, both positive and negative. Please also be respectful of one another. If you have a different opinion than someone, it is ok to share it but please be respectful. Please respect one another's privacy – what is said in this room stays in this room. Additionally, to protect your privacy, we will not be taking notes with names of who said what and we will not discuss what is said in these meetings with other focus groups, parents, or coaches.

At the end of our discussion, we will review some of the data we have measured thus far, some things you can remind your players that would help us with our research, and the contents of the coach reports that we hope to have your way by our next meeting. I also have a few surveys for you to complete.

Just a reminder – We will be recording this conversation to make sure we can capture everything. Please speak clearly and try not to talk over one another. I may ask you to repeat yourself, if needed. Please also try to limit distractions, like cell phones during the meeting.

If you need to leave for any reason to use the restroom or to take a phone call, please feel free to do so.

Okay, so we'll go ahead and get started.

1. Can you tell me about your experience playing or participating in football – as a player, parent, coach?

2. Why do you coach youth football?

3. What is your coaching philosophy?

4. How do you structure your weekly practices?
    a. Why do you structure them that way?

5. In your opinion, what are the benefits to a young person participating in youth football?
6. What role do you play as a coach in your athletes' lives?
7. Tell me about your relationship, as a coach, with the parents of your players.
8. What are the key points of sport safety you discuss with your players?
9. Have you ever discussed sport safety with the parents of your players?
    a. If so, what aspects of sport safety do you discuss with them?
    b. If not, why not?
10. In terms of safety, what are your concerns, as a coach, about your athletes on the field?
11. What are your hopes and goals for your team this fall?
12. What are you hoping to learn from participating in the focus groups?
13. Do you have any other thoughts related to what we've talked about today that you'd like to share?

Thank you so much for sharing your thoughts and opinions today! Here's the plan moving forward:

We will have three additional focus group meetings during September, October, and November. Our upcoming meeting topics will include common drills and contact scenarios in football, the structure of practice and how it relates to player development and player safety, as well as concussions and hits to the head in football. Additionally, we will discuss some of the biomechanics and video data we collect with the sensors on field.
