# Supplementary material for: Implementing injury prevention strategies in community-based youth football: The role of parents, coaches, and organizational leaders
Source: PLoS One. 2025 May 30;20(5):e0322373. doi: 10.1371/journal.pone.0322373 (PMC12124582; doi:10.1371/journal.pone.0322373)
Supplement: S4 File — (PDF) [file pone.0322373.s004.pdf]

Coach Focus Group #3 - Moderator's Guide

Good evening, everyone! Welcome back to our youth football parent focus group meetings. My name is Jill Urban. I am professor at Wake Forest University and I will be leading our discussion today.

As a reminder, we have a new project starting this fall to work collaboratively with a set of stakeholders in the local youth football community to create and test a practice structure to reduce head impact exposure while developing the skills needed to play football effectively and safely. To inform that effort, we would like to learn more about the perspectives of parents and coaches about football, while sharing some of the data collected on field. I'd like to introduce you to [Madi, Ty, Alexandra, Tina]. He/she is a graduate research assistant, and he/she will be taking notes today.

Today, we will be having a discussion about practice drills and coaching technique. Before we get started, I wanted to remind you of our ground rules. First, there are no right or wrong answers to my questions. We genuinely want to hear from you so please share your perspectives and experiences, both positive and negative. Please also be respectful of one another. If you have a different opinion than someone, it is ok to share it but please be respectful. Please respect one another's privacy – what is said in this room stays in this room. Additionally, to protect your privacy, we will not be taking notes with names of who said what and we will not discuss what is said in these meetings with other focus groups, parents, or coaches.

Just a reminder - I will be recording this conversation. Please speak clearly and try not to talk over one another. I may ask you to repeat yourself, if needed. Please also try to limit distractions, like cell phones during the meeting.

If you need to leave for any reason to use the restroom or to take a phone call, please feel free to do so.

I'd like to start today with a few questions from our previous discussion –

1. After reviewing the clips and seeing the biomechanics at our last meeting, what additional thoughts have you had about the contact you see in practices and games?

    a. What, if anything, has changed in the way you see contact on field?

2. Describe how you have structured your practice sessions this season and why.
    a. Has your week-to-week practice structure changed since we first met in August?

3. How have the coach reports informed your understanding of the head impacts of your athletes?
    a. How have they informed your understanding of how the drills influence head accelerations experienced by athletes?

4. Based on what we have gathered for your coach reports, the top five drills you conduct as a team are:

Team 1	Team 2
a. Team Scrimmage<br>b. Angle Tackle<br>c. Oklahoma<br>d. Inside Run<br>e. Individual	a. Team Scrimmage<br>b. 1 on 1 Block<br>c. Open Field Tackle<br>d. 1 on 1 Pass<br>e. 1 on 1 Tackle

5. For each drill:
    a. Describe the drill set up.
    b. How do you describe to your players what they will be doing or what they are supposed to do in this drill?
        i. Do you run moderated speeds of this drills to walk through the motions before conducting at full speed?
    c. What is the purpose of conducting this drill?
    d. What aspects of strategy, skill, or technique do you reinforce among your athletes during this drill?

[Review week to week trends in coach reports]

6. How do you talk to your athletes about the technique they use in games?
    a. Or how do you ensure that what you coach in practice is translated to the game setting?
        i. Or follow up when they don't use proper technique?
    b. How do you prepare or instruct technique or strategy to new players (0-1 years of football) compared to players who have been playing for a few years?
    c. How do you correct bad habits or poor technique among your players?
    d. Is there a point in the season when you shift the focus away from technique to strategy or something else?

7. From your perspective, what are your top three needs to effectively coach your team and why?

8. If you could do whatever you want (be king/rule the youth football world), and if resources were not an issue, how would you design a practice to meet the needs of your athletes?

    a. If you could do whatever you want (be king/rule the youth football world), and if resources were not an issue, how would you design a practice to best prepare your team to win?

Lastly, I have a few questions about your perspectives related to recent rule changes:

9. What are your opinions of recent rule changes in games in the NFL around head impact and concussion safety?
    - Targeting
    - Change in kick-off location (35% reduction in concussions after this rule change)
    - Prohibition of chop blocks, horse collar tackles, crackback blocks
    - Defenseless player protection, elimination of blindside blocks

10. What do you think about time limitations on the amount contact allowed in practice that have been implemented at many levels of play?
    a. Example: maximum of 15 minutes of live action contact per week at the high school level of play

11. What do you think about organizations banning high risk drills such as Oklahoma, Bull in the Ring/King of the Circle, half-line (NFL)?

12. What are the challenges in implementing or enforcing these types of changes at the youth levels of football?

13. What are you hoping to learn or discuss in our last focus group?

Thank you so much for sharing your thoughts and opinions today!

We will have one more focus group meeting in November. We will wrap up for the season, discuss opportunities and barriers to reducing head impact exposure in youth football, and discuss our plans to work with stakeholders over the next year.

-
