# Supplementary material for: Implementing injury prevention strategies in community-based youth football: The role of parents, coaches, and organizational leaders
Source: PLoS One. 2025 May 30;20(5):e0322373. doi: 10.1371/journal.pone.0322373 (PMC12124582; doi:10.1371/journal.pone.0322373)
Supplement: S5 File — (PDF) [file pone.0322373.s005.pdf]

Coach Focus Group #4 - Moderator's Guide

Good evening, everyone! Welcome back to our youth football parent focus group meetings. My name is Jill Urban. I am a professor at Wake Forest University, and I will be leading our discussion today.

As a reminder, we have a new project to work collaboratively with a set of stakeholders in the local youth football community to create and test a practice structure to reduce head impact exposure while developing the skills needed to play football effectively and safely. To inform that effort, we would like to learn more about the perspectives of parents and coaches about football, while sharing some of the data collected on field. I'd like to introduce you to [Madi, Tina]. She is a graduate research assistant, and she will be taking notes today.

Today, we will be having a discussion about the data we collected last fall. Before we get started, I wanted to remind you of our ground rules. First, there are no right or wrong answers to my questions. We genuinely want to hear from you so please share your perspectives and experiences, both positive and negative. Please also be respectful of one another. If you have a different opinion than someone, it is ok to share it but please be respectful. Please respect one another's privacy – what is said in this room stays in this room. Additionally, to protect your privacy, we will not be taking notes with names of who said what and we will not discuss what is said in these meetings with other focus groups, parents, or coaches.

Just a reminder - I will be recording this conversation. Please speak clearly and try not to talk over one another. I may ask you to repeat yourself, if needed. Please also try to limit distractions, like cell phones during the meeting.

If you need to leave for any reason to use the restroom or to take a phone call, please feel free to do so.

1. I am going to review a summary of the data we collected last fall. This will include the number of head acceleration events, percentage of head acceleration events that were associated with a hit to the head, head acceleration events per practice, per game, and more detailed information about head impacts in practice drills and game scenarios.
    a. Can you tell me a little bit about what you are expecting to see in the season summaries?
        i. Hits per practice
        ii. Hits per game
        iii. Percentage of head acceleration events that are head impacts
        iv. Drills
        v. Game scenarios

Review biomechanics data – [15 minutes]

1. Tell me what you think about the season summary.
    a. Does the data look as you had expected?
    b. Is there anything surprising to you?

2. You may know that we individually verify each mouthpiece recorded event to make sure that what we are reporting, is the result of a collision in football and not from them running or fiddling with the mouthpiece. We are currently working on methods to help us review the data more quickly. How frequently would you want to receive head impact data about your team during the season?

3. How would you use the information from the mouthpieces to inform activities for your team in practice?

4. If you had information about individual athletes, how would you use that information as a coach?

5. How do you think coaches could use the data we collect to improve safety or performance of athletes?

6. As a coach, what would be helpful to know about head impacts and concussions BEFORE the season starts?

7. In your opinion, what is the greatest opportunity to reduce hits to the head in practice youth football?

8. What are the challenges in implementing changes to practice at the youth levels of football?

a. What are the challenges in enforcing changes to practice at the youth levels of football?

b. What are the challenges in sustaining changes to practice at the youth levels of football?

9. In your opinion, what would facilitate or motivate coaches or league officials to engage in discussions about changes to practice at the youth level of football?

10. From your perspective, how do you define a successful football season for your team?

11. What are the top three things that contribute to your team's success in football and why?

12. Do you have any thoughts or questions about what we discussed today?

13. What are you hoping to learn at the conclusion of our study?

Thank you so much for sharing your thoughts and opinions for this project!
