# Supplementary material for: Implementing injury prevention strategies in community-based youth football: The role of parents, coaches, and organizational leaders
Source: PLoS One. 2025 May 30;20(5):e0322373. doi: 10.1371/journal.pone.0322373 (PMC12124582; doi:10.1371/journal.pone.0322373)
Supplement: S6 File — (PDF) [file pone.0322373.s006.pdf]

League Official (i.e., Organizational Leader) Interview #1 - Moderator's Guide

Good afternoon, everyone! Welcome to our first youth football coach focus group. My name is Jill Urban. I am an Assistant Professor at Wake Forest University. I have worked with several youth sports organizations, including your organizations studying concussions and head impacts in youth football for the past 9 years and have truly appreciated the partnership with the league for the last several years. I am also a parent myself with four-year old twin boys.

We have a new project to work collaboratively with a set of stakeholders, including coaches, players, and officials, in the local youth football community to create and test a strategy to reduce head impacts and concussions while developing the skills needed to play football effectively and safely. We collect a lot of data that may be useful for coaches, so we would like to put the data back in the hands of the decision makers to try to create an effective practice environment while minimizing the risks, where possible. To inform that effort, we would like to learn more about the perspectives of parents, coaches, and organizational leaders about football, while sharing some of the data collected on field in our meeting.

What we'll be doing today is having a discussion about your experiences and perspectives being a leader of youth football organization. I will be asking several questions and facilitating the discussion – none of them are hard. Before we get started, I wanted to state a few ground rules. First, there are no right or wrong answers to my questions. We genuinely want to hear from you so please share your perspectives and experiences, both positive and negative. To protect your privacy, I will not be taking notes with your name, you don't need to say your name or organization in the recording, and we will not discuss what is said in these meetings with other focus groups, parents, or coaches.

Just a reminder – We will be recording this conversation to make sure we can capture everything. Please speak clearly.

If you need to leave for any reason to use the restroom or to take a phone call, please feel free to do so.

Okay, so we'll go ahead and get started.

1. Can you tell me about your experience playing or participating in football – as a player, parent, coach, league official?

2. Can you tell me about your role in your organization?

3. What brought you to this role or why did you choose to take this role in the organization?

4. What is your leadership philosophy?

5. In your opinion, what are the benefits to a young person participating in youth football?

6. What role do coaches play in your athletes' lives?

7. Tell me about your relationship with the coaches in your organization.

8. Tell me about your relationship with the parents in your organization.

9. What are the key points of sport safety you discuss with your coaches?

a. How do you communicate requirements or expectations around safety to your coaches?
b. How do you influence your coaches to adopt different practices or consider different coaching approaches in practice?

10. Have you ever discussed sport safety with the parents of your players?

    a. If so, what aspects of sport safety do you discuss with them?
    b. If not, why not?
    c. How do you communicate organizational requirements or expectations around safety to families in your organization?

11. In terms of safety, what are your concerns, as a league official, about your athletes on the field?

12. What are your hopes and goals for your organization this fall?

13. What are you hoping to learn from participating in the next interview?

14. Do you have any other thoughts related to what we've talked about today that you'd like to share?

Thank you so much for sharing your thoughts and opinions today!
