# Supplementary material for: Implementing injury prevention strategies in community-based youth football: The role of parents, coaches, and organizational leaders
Source: PLoS One. 2025 May 30;20(5):e0322373. doi: 10.1371/journal.pone.0322373 (PMC12124582; doi:10.1371/journal.pone.0322373)
Supplement: S7 File — Codebook used in this study. (PDF) [file pone.0322373.s007.pdf]

Evidence-Based Intervention in Youth Football Codebook

Code	Definition	Exclusion criteria
Adolescents	Mention of adolescents, kids, and/or 11-13 year olds	
Aggressive behavior	References to anger, agression, or related behaviors	
Aspirations	A response stating the hopes and goals for the team, athlete, or son	
Athlete experience in the sport	Responses referring to athlete experience (ie how long an athlete has played football)	
Attendance, tardiness	Responses related to attendance, tardiness, having all kids present	
Barriers to change	Response describing challenges or barriers to implementing changes in youth football	
Benefits of youth football and/or team sports	A response supporting or indicating the benefits of youth football and/or team sports	Downsides or cons of participating in youth football or sports
Biomechanics	A response referring to the biomechancis data collected from athlete; may also refer to it as concussion sensors, head impacts or head impact sensors, mouthpieces, or may just refer to it as "the data"	

Coach to athlete communication	Descriptions of how the coach communicates with the athletes	
Coach to coach communication	A response discussing communication among coaches; this may include how they communicate, or how their communication plays a role in their coaching	
Coaching challenges	Responses indicating challenges in meeting coaching expections	
Coaching philosophy	A participant states their personal theory or attitude about coaching, or instructing youth athletes; may also include coaching strategy	
Concerns about athlete safety	A response that specifically states a concern about athlete safety	concerns unrelated to safety
Concussion	Anytime someone mentions concussions	
Connection to the sport	A participant mentions how they are connected to the sport of football; through personal or familial participation	

Descriptions of football	A response the describes the nature of the sport
Enthusiasm for the study	Responses indcating enthusasm for the study or for being part of the study
Existing rules	References to existing rules in youth football or other levels of play
Expectations of coach	Responses that indicate parents' expectations of coach or presumptions about how the coach be.
Football translating to real life	Specific responses related to experiences and training on the field translating to life off the field
Gaps in knowledge	Responses that indicate the participant wants to know more about something, or has a question or curiosity - gaps in knowledge
Governing bodies	Responses that mention organizational decision making or governing bodies that have influence over youth football
Influences on adolescents	Responses that describe how the NFL, older players, their family, or other figures may influence their behavior on field or decisions around participation.
Interest in knowing more	Responses that state something the respondents want to know more about

Interest in research outcomes	Responses indicating interest in what would come of the project - as it relates to football safety, information for coaches, etc	
Love of the sport	A participant specifically states their "love" of the sport; this code would also include reasons why they love the sport	
Mental focus	Response related to mental focus; may inlcude its role injury or injury prevention	
Needs of coach	Response indicating needs of coach to effectively or successfully coach his team	
Other concerns	A response that states a concern about athletes or the team, unrelated to safety	concerns related to safety
Parent-Coach relationship	Statements describing the parent - coach partnership, communication, relationship, etc.	
Parent perceptions of athlete	Responses indicating what a parent thinks their child knows; may include reference to what they know about hits or concussion	
Peer Support/Friendship	Statements describing friendship, comraderie, support from peers as it relates to football	

Perceptions of contact	A response descibing or speculating how often or how hard a player gets hit on field
Personal injuries	Participant giving example of injury they experienced themselves or observed from others, including their kids or other athletes in sports
Positive coach feedback	Positive affirmations, or positive comments from parents about coaches; also includes comments from coaches positively affirming other coaches or seaking positive feedback for themselves.
Practice structure	A response describing what is done in practice or how practice is structured
Purpose of drills	Descriptions of drills included in practice, including how and why the drill is conducted
References to film	Responses describing how film or video are used for football among athletes, coaches, and/or parents

Risks of other sports	Responses that point out the risks of other sports	
Risks of youth football	A response indicating the risks or downsides of participating in youth football or sports	Benefits of youth football or sports
Role of coach	A response stating the role of the coach in football	
Role of practice	A response describing the role of practice for the team and/or athlete skill development	
Sport safety discussion points; warnings	A response indicating the aspects of sport safety that are communicated to athletes by stakeholders (parents or coaches).	
Suggested changes to football	Responses from parents and coaches suggesting ideas for changes to football	
Stigma around football	Responses indicating what other people think about the sport	

Team	References to team, teamwork, for groups of people working together
Technique	Responses related to player technique
Toughness in football	Responses related to toughness in football, may include recognizing pain vs injury
Why son plays football	Responses stating why son plays football

Example

But, you know, and, you know, just building the relationships with, with kids, you know, that it's, it's funny how 12 year olds respond to people who treat them like people. So we try to talk to them and treat them like young men. And, you know, there's discipline that's involved a lot of times, of course they're 12, but you know, that's our philosophy is we we coordinate as a group and the players, I think, understand that we're all on the same front when we bring something to them.

My son needed a way to release a lot of pent up anger and aggression, without beating up his cousins. So yeah, This was the most constructive way to do it. Like I'm not putting my son in boxing. That's not happening.

Just to get better every week, every week, every practice, get a little bit better than you were. And I believe that that is the goal, and the wins will come.

And I had already told him that at the beginning, my son is gonna be a little slow. He's because he's coming from not playing really no football. But, you know, just patience. And I think that the coach has a lot of patience. He explains everything.

We try to stay on schedule. And like I said when kids don't show up on time, it knocks everything off but now we still can go based on all this practice plan to where it's one we can work individually as coaches to get our kids ready in these drills, but also for the game also as well.

it's just that you know you probably got to get to some of these old school coaches who don't want, who don't want change. Yeah, so that's probably gonna be fresh, fresh bodies fresh coaches fresh players over time it'll help if we keep doing stuff like this.

The physical activity definitely. And then, well, the whole life team thing, you know, learn to work as a team. Like being good or bad, you know, affects everybody, not just them. If somebody messes up, everybody suffers. Like they learn little lessons in there.

I'm excited to see to like, learn more about the data and see how we hear you know, we've been hearing for years and decades about, you know, how the head injuries, I'd like, to look at that, as well.

pretty much other teams and you know, their, their kids will like drop and then, "get up" as many of yell at them, get up and up, throwing them out there like they're rag dolls. This one's not like this is like, they're good.
We communicate mostly through text and like, like most things we take a lot of things get lost in translation. And we forget what we're saying, you got to scroll back through. You know, so yeah, so being able to have the face to face contact, then building that camaraderie outside of the field as you get to learn a lot more about people

Speaker 1: You have kids that have been playing for five or six years with each other, kind of went through this recently with a kid whose his body changed. He was a quarterback when he was six. He's obviously not a quarterback anymore, just looking at his body and his movement skills. But him, his parents, everybody had a hard time accepting that because he was a quarterback since he was six. But as coaches we knew, from especially during that COVID year, his body changes, he's sitting around a lot, gained weight. He's not a quarterback anymore. He didn't want to accept it so he left the team. And his parents didn't want to accept it. But it's those decisions we have to make as coaches because we have to put them in the best position to succeed and to... Speaker 2: protect himself.

Coach: But my biggest thing is as a coach, I don't care about wins, I don't care about losses. What I care about most. And these guys take what we teach them on the field and transposing that into life being better human beings. And so the only way they can do that is they learn how to be disciplined on the football field, they give effort, and don't quit on themselves.;

My biggest concern is blindside blocks. Because, I mean, I've seen some people get. I've seen that I've seen some bad blindside blocks that people just don't recover from easily. I mean, weeks without playing, practicing, or anything. So yeah, that's, that's my biggest concern is the blindside block. Because if you standin' around someone is always looking for the weak deer to shoot. And if you're that guy, you gonna get knocked out, you're gonna get knocked out... completely.

I don't have the conversation about concussion. I don't know the symptoms, I just, if my son has anything to do with a concussion, he's probably given a couple.

Family. A lot of family. I played two years Junior College and a little bit in high school.

It's football is a violent sport.

Well you can't do Oklahoma no more based on the level of impact. So now we got to shorten the distance on the point of contact that teach the technique to get the kids better so when it comes game time, they know when to break down actually strike a player versus just running through the body where it causes that impact that pains, as well,

And so we have always kind of said, like, we're gonna make sure that you have you're on a team where the coaches are doing the right thing and know what they're doing.

I don't care about the football field, I want to see you succeed off the field.

Example 1: Yes, I would like to learn do's and don'ts as far as drills and technique. And, you know, just you know, keeping kids safe because not only the other kids that I've built a rapport with my son, like my son, plays football, he loves football, and I don't want to see anything happen to my son. So I would like to learn do's and don'ts; Example 2: I want to see how I can make our practices more safer, and also in the game also as well, because like I said, at a young age right now, their bios to growing is still maturing as young adults. And so I want to look at the impacts long term, to see how can we prevent. I want to see how we can prevent these things long term.

I think football is one place where the safety has started from the top, and it's coming down like they've implemented the safest practices at the NFL level, and then the college level and it takes longer for it to get to the youth level because the people coaching played in a different era of football.

And, you know, we, I always say, you know, sports center a lot of times is their greatest enemy. Because they see these big hits, and they see their favorite player blowing somebody up, and they think I gotta do that every play. At line, that rarely happens that you know, as a player, especially on the line, you know, you got to get hit every single play.

I'm excited to see to like, learn more about the data and see how we hear you know, we've been hearing for years and decades about, you know, how the head injuries, I'd like, to look at that, as well.

...but it's a gladiator sport. That's why people love it so much. Yeah, cuz I'll say my son since the age of six, when it comes to football. He flips a switch on, and it's hard to get him to refocus. Because when he steps on those lines. You're going to either get frantic like full-go like animal or you're going to get him goofing off on the sidelines. ;

Top three time, And it's only two important... or two and a half. You need time, and you need willingness from the players and coaches, I mean not players and coaches. Players and parents

I'm concerned about my son missing a block.

And so it's just communication that goes outside just the coaches and players, that with the parents too. I think we have we have the parents trust us, this group is probably the best group of parents I've ever coached with. I mean, they're not down on the field yelling and screaming at us, they half the time they drop their kids off, and know they're in good hands, and they come back two hours later, they go shopping and do what they need to do for the day. So, you know, it's, it's about, it's about the whole community. Because we're not we're not people look at coach like, coaches, coaches aren't just coaches, we help we're helping raise kids, you know, so we have to be good role models, too. And teach them the right things he said, we're not developing just players, like he said, we're developing young men too

So my son definitely knows what to look for when he gets hit

My son plays baseball, and he's played basketball and football. So he's pretty much only done team sports hasn't been any individual sports really. So I would say that's the majority of his free time is playing sports, either on an organized team or out in the yard with friends or whatever.

I mean, you know, It's evitable, I mean, when you're playing offense and defensive line, I mean, the first 90% 90% of plays the first contact is the forehead played on the helmet. I mean, just because you have two kids lining straight up in front of each other, and they're both coming up this way. So it's either they're here or the face mask touch, but they're a foot apart. So it's not a huge impact.

I poured right to go to a play wasn't paying attention. I got hit right here on the side got a concussion. Not only did I get a concussion, I got hit so hard that it jarred these fatty tissues of the spine of my neck, and created a fluid sack on the back of my neck but it's still there to this day. I've had MRIs, I've had CAT scans, nothing they can do about it, I can say it's just literally fat tissue that just jarred away. So I deal with that for the rest of my life by not really having true technique at that time.

t. And the coaches do as well. I think they do since my son has been playing with the Panthers since he was younger. I feel like they've always done a really great job. And when someone gets hurt, or they think someone got hit too hard, making sure that they are ok

The Monday, then Tuesday would be then we go a team, I guess go over team concept with defense on Monday, Tuesday will be offensive day. Individual, you know, putting in plays, and then we do sometimes have a light scrimmage. But we do, we don't tackle to the ground after Monday. It's just wrap up so the running back's down when you wrap up. So there's a there's a little contact but it's not full contact. Thursday would be same thing. Depending on what we need to brush up on for Monday and Tuesday. Game prep and special teams so you're kicking games, punting, field goal, kick off.

When you're playing when you're playing on Saturday, you know, any day Thursday Friday Saturday Sunday you know whatever level you play when you line up it's me versus you. And my job is to beat you. Before I can do anything else so like, that's our one on one drills of stressing the fact that you have to do your job and right now he's your job. And if you don't beat him right now. How can we assume or trust that you're gonna beat that person on Saturday? Right now, so it's really, that's our probably our most physical drill is the, you know, it's not tackling to the ground but it's the one on one hand fighting the dog eat dog in the trenches.

It can happen in any sport doesn't have to be football. My son had one in basketball.

you know, so it was just this one has the potential sometimes to be a little more dangerous

Parent: Yeah, like it's just, just with all that, you know, I wish we could have had, you know, six or seven years of him playing football, I wish I had some more of Coach Dennis, because when you have a coach this teaching you the game at the next level before you even get to the next level. And it's just like, I knew my son know football but it was just that when he explained the plays with me when we go over them, I'm like, How'd you get that? I mean, and just with watching, he can enjoy when we watch it as a family and stuff so with, you know it's good to have that responsibility from the importance of having a good coach, knowing that your grades are good, and you know organization, You know, to make sure the kid is doing what they're supposed to do, because that's what you want.

Every every hitting drill every every time we're having contact. No matter who's getting up in the line. I always say remember, keep your head out of play, head out of the play, head out of the play head out of play, don't use your head, head up, head out of play.

Yeah. well that's what I wonder if like, because you have kids coming into it at different ages. So like you have a 12 year old who's played for years and 12 year olds who are new to it, like, would there be a benefit to starting a season with like, just a technique camp, rather than going to like individual practices where coaches are doing drills? But like

there's a stigma around football like, a lot of people. I'm sure you guys talk to moms and, and oh no, my son is not playing football, no. And this one's like, no, I actually want him to play football. She's like, no, you're not playing. But like they're just it's so unsafe and all of these things.

Example 1: Yeah, because if they do then, with football being the ultimate team sport with 11 people being on one side, if everyone's getting better and pulling in the same direction. We don't have to be that good to beat people. ; Example 2: It's the ultimate team sport, there's a position for every shape and size kid. And 11 guys on the field have to all be doing the right thing for it to work. So it teaches teamwork and responsibility.

Example 1: And so what happens is a lot of things that keep that goes a lot of injuries, is when you're out of control when you're, when you're so mentally, like you're amped up, you lose all technique you lose all abilities everything you're taught, that you're coached for, and then that's when you get hurt, and I've seen it happen to a lot of kids whereas they, they're in that that zone where zoned out, and they're just doing their own thing, they don't take the coaching and they get hurt, and I've seen it time and time again.; Example 2: But I do bring up technique, because I think that's where safety starts.

Yeah, you're gonna have, you're gonna have those pains because you won't be.. when the season start, even the spring, even though we had a big break, but this is two years since we played a full fall season. So, you know, that's not enough preparing on the pain that you're going to feel knowing that you play every Saturday. But that is something that I will get through the season.

(Example 1) They just love it. It's just fun for them, I guess. ; (Example 2) My son has loved football since he was five. So it's all about football, yeah.
